# Design and Validation of a Droplet-based Microfluidic System To Study Non-Photochemical Laser-Induced Nucleation of Potassium Chloride Solutions

**DOI:** 10.1021/acs.cgd.3c00591

**Published:** 2023-07-19

**Authors:** Vikram Korede, Frederico Marques Penha, Vincent de Munck, Lotte Stam, Thomas Dubbelman, Nagaraj Nagalingam, Maheswari Gutta, PingPing Cui, Daniel Irimia, Antoine E.D.M. van der Heijden, Herman J.M. Kramer, Hüseyin Burak Eral

**Affiliations:** † Process and Energy Department, Delft University of Technology, Leeghwaterstraat 39, 2628 CB Delft, The Netherlands; ‡ Department of Chemical Engineering, KTH Royal Institute of Technology, Teknikringen 42, 114-28 Stockholm, Sweden; § School of Chemical Engineering and Technology, State Key Laboratory of Chemical Engineering, Tianjin University, 300072 Tianjin, People’s Republic of China

## Abstract

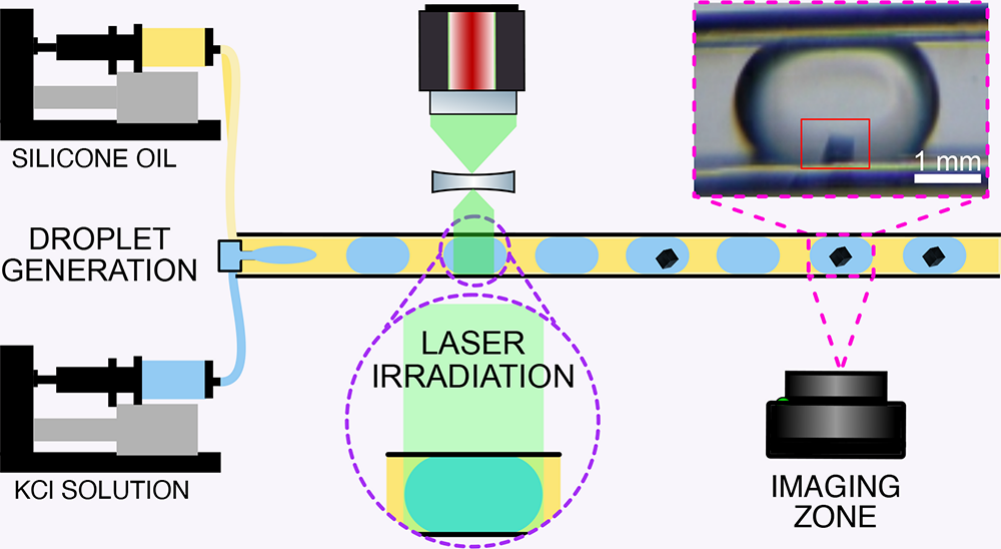

Non-photochemical laser-induced nucleation (NPLIN) has emerged as a promising primary nucleation control technique offering spatiotemporal control over crystallization with potential for polymorph control. So far, NPLIN was mostly investigated in milliliter vials, through laborious manual counting of the crystallized vials by visual inspection. Microfluidics represents an alternative to acquiring automated and statistically reliable data. Thus we designed a droplet-based microfluidic platform capable of identifying the droplets with crystals emerging upon Nd:YAG laser irradiation using the deep learning method. In our experiments, we used supersaturated solutions of KCl in water, and the effect of laser intensity, wavelength (1064, 532, and 355 nm), solution supersaturation (*S*), solution filtration, and intentional doping with nanoparticles on the nucleation probability is quantified and compared to control cooling crystallization experiments. Ability of dielectric polarization and the nanoparticle heating mechanisms proposed for NPLIN to explain the acquired results is tested. Solutions with lower supersaturation (*S* = 1.05) exhibit significantly higher NPLIN probabilities than those in the control experiments for all laser wavelengths above a threshold intensity (50 MW/cm^2^). At higher supersaturation studied (*S* = 1.10), irradiation was already effective at lower laser intensities (10 MW/cm^2^). No significant wavelength effect was observed besides irradiation with 355 nm light at higher laser intensities (≥50 MW/cm^2^). Solution filtration and intentional doping experiments showed that nanoimpurities might play a significant role in explaining NPLIN phenomena.

## Abstract 2

The article describes a droplet-based microfluidic setup to study NPLIN with the droplets containing crystals identified using the deep-learning method. Experimental parameters such as solution supersaturation, laser wavelength, laser intensity, solution filtration, and intentional doping of nanoparticles are studied using supersaturated aqueous KCl solutions to quantify its influence on nucleation kinetics and draw parallels to the proposed underlying NPLIN mechanisms in the literature.

## 1 Introduction

Crystallization is arguably the most widely used separation and purification techniques applied in a multitude of industries such as pharmaceuticals, food and beverage, agriculture, fine chemicals, and many more.^1−8^ The process of crystallization consists of two main stages, namely nucleation and growth. Significant advances in the understanding of the mechanism of nucleation from solution have been made,^9−13^ yet many aspects of the nucleation process, such as the mechanism of polymorph selection and on-demand spatial–temporal control, are far from being completely understood. This makes the deterministic design and scale up of industrial crystallization processes challenging.

In an attempt to improve control over nucleation and consequently over crystal properties, more advanced crystallization methods are sought. One promising technique is non-photochemical laser-induced nucleation (NPLIN), where a nanosecond laser pulse is used to trigger instantaneous crystallization in supersaturated solutions that would otherwise take several weeks to nucleate without any external interference.^14^ This physicochemical process is termed ‘non-photochemical’ because the solution does not absorb any light at the irradiated wavelength, and hence the laser pulse does not induce any photochemical reaction.^15^

Numerous studies have been conducted on this phenomenon, gathering data on experimental parameters influencing NPLIN such as laser intensity, laser polarization, supersaturation, and impurities.^15−18^ Many compounds, including small organics,^19−21^ metal halides,^22^ single-component systems,^23,24^ dissolved gases,^25,26^ and a macromolecule – lysozyme,^27^ have been crystallized with NPLIN. Based on the collected observations, three mechanistic hypotheses were proposed to explain the NPLIN phenomena. The first mechanism is based on the optical Kerr effect (OKE), i.e., the electric field of the laser induces a dipole moment in the system and can further produce a torque to align the molecules in the cluster along the field direction accelerating the structural order in the cluster to form a crystal.^14^ This light-induced alignment of the molecules has also been proposed to explain reports on polymorphic form control with polarization of light, reported for supersaturated solutions of glycine,^28^ sulfathiazole,^29^ and carbamazepine.^30^ The second mechanism was proposed by Alexander and Camp^16^ who suggested an explanation based on the isotropic electronic polarization (IEP). The hypothesis is based on the fact that, in the presence of an applied optical electrical field, the free energy of a dielectric particle is reduced when immersed in a medium of lower electric permittivity. The reduction in free energy of the pre-nucleating clusters leads to a reduction in the size of critical nuclei and thus enhances the nucleation kinetics. However, this mechanism fails to explain how NPLIN favors the preferential formation of certain polymorphs in NPLIN experiments. The third potential mechanism proposed is based on the heating of impurity nanoparticles existing in the system – molecular impurities (intrinsic) and/or dust particles (extrinsic). The nanoparticles are hypothesized to heat up on absorbing the incident laser light, and the resulting heat is then transferred to the surrounding liquid vaporizing volume of liquid around them. Upon evaporation of liquid, the growth of the vapor bubbles promotes the aggregation and accumulation of the solute molecules at the vapour liquid interface driving them to nucleate and form crystals. Yet, no clear consensus on mechanism has been reached as the proposed mechanisms fail to fully describe all the reported experimental results in the literature.^15^

Research on NPLIN is largely hindered by the stochastic nature of the phenomenon, requiring a substantial number of repeated experiments to draw definitive conclusions. Therefore, past research on NPLIN studies often used large numbers (order 10–100) batch samples to reach statistically significant data points, a labor-intensive procedure.^18,31^ In 2014, Clair et al.^32^ developed the first high-throughput controlled setup for NPLIN studies. The setup used an automated carousel holding 90 HPLC vials that a laser could irradiate through the air/liquid interface. Even though this setup takes away much of the manual labor, it still results in long processing times needed to obtain large data sets because of manual crystal detection. Microfluidics represents an alternative to acquire automated and statistically reliable data and has already proven its value in the investigation of crystal synthesis of pharmaceuticals, nanocrystals, and proteins.^33−40^

So far, only two studies on NPLIN in continuous systems have been reported in the literature. Hua et al.^41^ presented a single-phase microfluidic device that exposed a continuously moving supersaturated solution of KCl to pulsed laser beams. In their device, supersaturation is regulated by strict temperature control of the microchannel, which permits cooling of the solution upon entry and reheating near the exit to avoid clogging of the channel. This study provided insight into the effects of supersaturation, laser energy, pulse duration, and the number of pulses on the number of crystals and their size. The authors further expanded their work with their setup to study NPLIN on supersaturated aqueous glycine solutions.^42^ Upon irradiation of freshly prepared supersaturated glycine solutions (*S* = 1.4–1.6), no NPLIN effect was observed. However, a significant increase in nucleation probability was seen when the glycine solutions were left to age for 24 h in a sealed syringe. The effect of ageing glycine solution had already been reported in experiments conducted with milliliter size vials.^18,43^ Moreover, results of Hua et al.^41^ also agreed with prior batch studies that observed a change of glycine crystal morphology with increasing supersaturation.^20,44^

In this study, we present a droplet-based microfluidic setup tailored for NPLIN studies where the droplets containing crystals were identified using the deep-learning method. Using this tailor designed setup, we performed a systematic study of NPLIN-affecting parameters (laser wavelength, peak laser intensity, solution supersaturation, solution filtration, and intentional doping with nanoparticles) on supersaturated aqueous KCl solutions. The microfluidic device was designed to create stable, supersaturated droplets of the solution with desired volume, allowing every droplet to act as a separate micro-reactor. In comparison to the traditional manual methods used in NPLIN experiments which only allow for a limited number of experiments (10–100),^16,32^ our device enables the collection of a much larger quantity of independent data points, typically over 1000 experiments, effectively addressing the stochastic nature of the crystallization process. The NPLIN experiments are conducted by exposing aqueous KCl droplets of designated supersaturation (1.05 and 1.1) created by cooling the droplets from 40 °C to room temperature. The droplets are exposed to continuous 10 Hz laser pulses at designated peak laser intensity (varied between 10 and 100 MW/cm^2^) and wavelength (1064, 532, and 355 nm) with an unfocused laser beam diameter of 1.35 mm. Moreover, we report how filtration and addition of nanoparticles influences the NPLIN probability and discuss our results in the context of dielectric polarization and the nanoparticle heating mechanisms proposed for NPLIN.

## 2 Experimental Methods

### 2.1 Material

KCl (Sigma Aldrich, molecular biology ¸ 99.0 %, CAS: 7447-40-7) solutions in Ultrapure water (ELGA Purelab, U.K., 18.2 MΩ cm) and silicone oil, with a viscosity of 10 cSt (Sigma Aldrich, CAS: 63148-62-9), were used, respectively, as dispersed and continuous phase. The solutions were prepared by adding the designated amount of KCl to reach desired supersaturation at room temperature and stirred rigorously. The prepared solutions are then placed in an oven at 50 °C to ensure the complete dissolution of all crystals. The solutions were maintained at this temperature until they are used in experiments. Supersaturated solutions were prepared based on 352.4 g KCl/kg water solubility at 25 °C.^45^

### 2.2 Solutions Doped with Nanoparticles

In order to produce supersaturated solution samples doped with known amounts of solid nanoparticles, a stock solution of KCl with concentration *C* = 5.42 mol/kg (*S* = 1.127) was prepared and filtered into cleaned beaker at 50 °C. A known quantity (1.25 g) of liquid dopant was added to the filtered solution to give a resulting concentration of *C* = 5.29 mol/kg (*S* = 1.1). The liquid dopant included aqueous dispersion of iron oxide nanoparticles (≥97%, CAS: 1317-61-9, 50–100 nm nominal diameter), with pure water as a control, prepared in the similar way as given in the article from Ward et al.^46^ Dispersion was then subjected to ultrasonic treatment (750 W, CV334) for a period of 2 h before use to ensure maximum dispersion.

### 2.3 Microfluidic Setup

**Figure 1:**
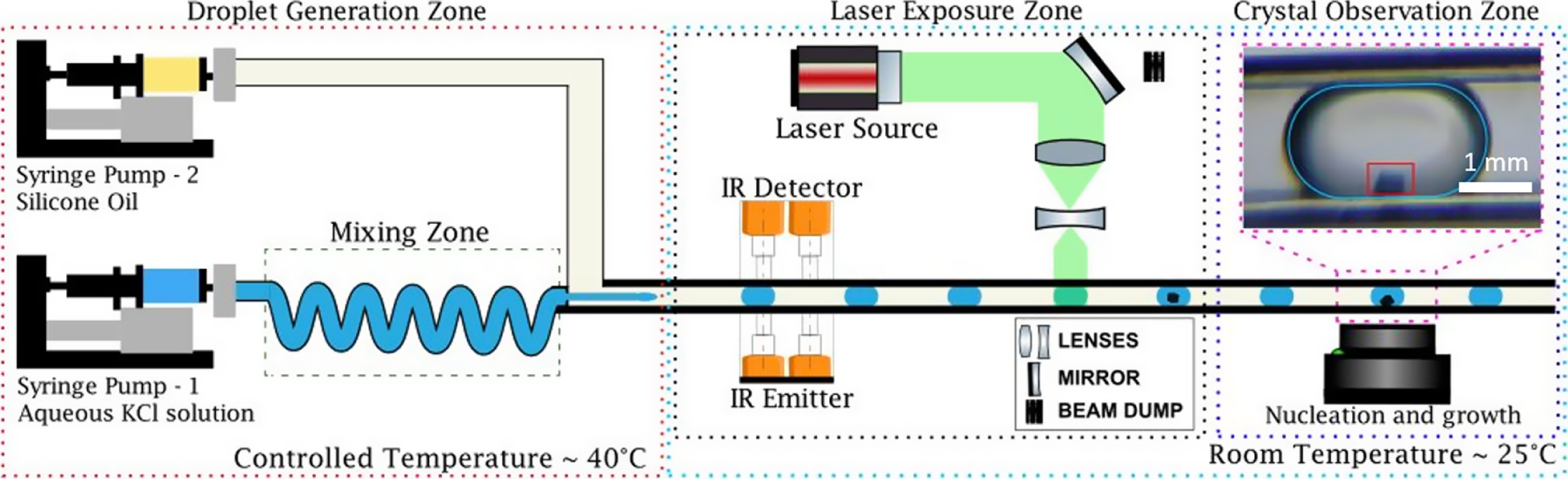
Schematic representation of the droplet-based microfluidic system designed for this study. The system consists of three different zones: droplet generation, laser exposure, and crystal observation.

A droplet-based microfluidic system to study NPLIN was designed and developed to generate large data sets (≈1000 droplets) for each parameter investigated, where each droplet acts as an independent crystallization reactor. The schematics of the system are shown in Figure 1. The system is divided into three main sections: the droplet generation zone, the laser exposure zone, and the crystal observation zone.

#### 2.3.1 Droplet Generation Zone

The droplet generation zone is placed within a temperature controlled environment, kept at 40 °C, to ensure that no crystallization takes place during the droplet generation process. Two microfluidic syringe pumps (NE-1002X-ES, New Era Pump Systems Inc.) are used: one for the dispersed phase, namely aqueous KCl solution, and one for the continuous phase, i.e., silicone oil. Both dispersed and continuous streams flow through polytetrafluoroethylene (PTFE, 900 μm diameter) tubes connected to the syringes, at 10 and 100 μL/min, respectively. Immediately after being pumped into the system, aqueous KCl solution encounters a mixing zone of 10 bends to ensure homogeneous solution concentration. Bends are reported to break the symmetry in the velocity field within the fluid direction by promoting variations in wall drag forces, thus inducing passive mixing.^47,48^ After leaving the mixing zone, the dispersed phase meets the continuous phase at a T-junction, for the coaxial formation of the droplets. The dispersed phase flows through an inner round capillary (Vitrocom Inc., borosilicate, 700 μm diameter) surrounded by a squared glass capillary (Vitrocom Inc., borosilicate, 900 μm side) through which the continuous phase flows, leading to the formation of the droplets at the edge of the inner capillary.

It is worth noting that both material and geometry changes, from round PTFE tubes to glass squared capillary, were necessary. PTFE tubing is not suitable to withstand the incident laser light while the square geometries help minimize reflection and refraction of the laser. The glass capillary was hydrophobized (see Supplementary Information Section S1(49)) to minimize the interaction between the droplet and square capillary, which could otherwise induce crystal nucleation within the droplets.

#### 2.3.2 Laser Exposure Zone

As the droplets form and flow through the square glass capillary, they enter the laser exposure zone, located outside the temperature-controlled environment (40 °C). Hence, droplets undergo cooling to room temperature (25 °C) and become supersaturated after travelling approximately 15.6 mm, a distance much smaller than the distance between the T-junction and location of the laser irradiation (see Supplementary Information Section S2(49) for details of this calculation). An infrared (IR) sensor set was implemented at the beginning of the laser exposure zone. Data from the set of IR sensors are used to count and measure droplet velocity and volume.

Droplets are irradiated 8 cm after leaving the temperature-controlled environment by an unfocused pulsed laser beam (10 Hz, 9 mm diameter, Nd-YAG laser, Continuum Powerlite DLS 8000). The beam was redirected toward a set of two lenses, positioned in a telescopic fashion, by the first mirror, as shown in Figure 1. In this arrangement, the reduction of the beam size (from 9 to 1.35 mm diameter) and amplification of the laser intensity are achieved. As the droplets are irradiated 8 cm after leaving the temperature-controlled environment, a distance much greater than 15.6 mm predicted to reach desired saturation, we can safely assume that the droplets are irradiated after they reach the designated supersaturation.

#### 2.3.3 Crystal Observation Zone

The observation zone is located 16 cm after the droplets were exposed to the laser beam. Within this distance, KCl crystals can nucleate and grow within the droplets. Droplets in the capillary are imaged using an objective lens (4X, 0.1 NA), a microscope camera, and a diffuse white LED light source. The observation time here, limited by the length and cross-sectional area of the squared glass capillary, positioning of the imaging system, and flow rates of continuous and dispersed phases, was found to be approximately 70.7 s. Droplets containing crystals were counted manually and automatically through a tailored image processing code for comparison. The results of automatic count of droplet containing crystals were used in evaluating the cumulative nucleation probability at a fixed time lag of 70.7 s, defined here as the ratio of the droplets containing crystals to the total number of droplets for a given experiment.

### 2.4 Droplet Identification

#### 2.4.1 IR Sensors

**Figure 2:**
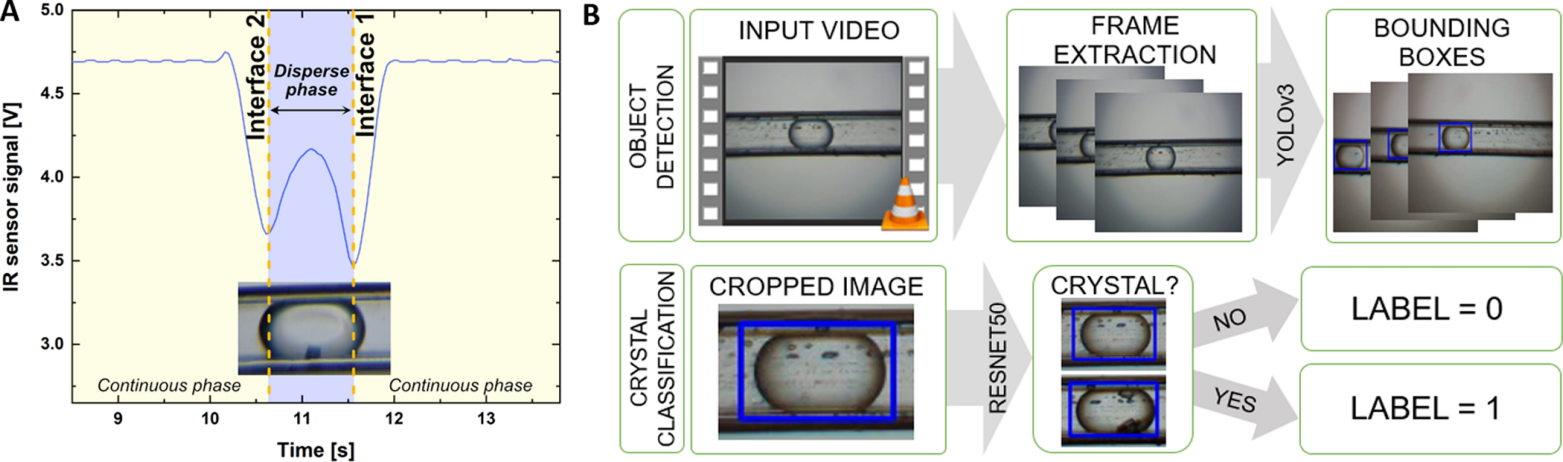
Droplet identification using (A) infrared (IR) sensors and (B) illustration of deep-learning method implemented to calculate cumulative nucleation probability automatically.

The IR sensors were used as a non-invasive measuring technique to detect interfaces between the continuous and the dispersed phase through the refracting and reflecting nature of the curved interface between them. Each IR sensor consists of an IR LED and photodiode (BPV10NF), located on opposite sides of the capillary. Both parts were held in place by a 3D-printed sensor holder and mounted in a circuit with two operational amplifiers (Op-Amp MCP6241) to improve signal quality. The recognition of the droplets by the IR sensors is based on the differences in light transmission from the LED to the diode at the edge of the oil water interface of the droplets compared to that in the continuous phase respectively. Since the curvature of the interface deviates the light emitted by the LED, fewer photons reach the photodiode and a drop in the voltage generated can be seen. The sensors were connected to a hardware prototyping platform (Arduino Mega, ATmega 328P, Arduino LLC, Ivrea, Italy) that provided data collection and analysis. The data were used to identify the peak of each voltage drop, i.e., the liquid–liquid interfaces, and to count and estimate the volume and determine droplet velocity. An example of data collected by Arduino can be found in Figure 2A.

#### 2.4.2 Deep-Learning Method

Parallel to the IR sensors, another droplet identification technique was developed to count the droplets, estimate their length and determine their velocity from experimental videos using deep-learning method. In addition, the algorithm developed was also capable of counting the droplets containing crystals automatically. The algorithm includes object detection and crystal classification based on two deep-learning models as shown in the flowchart in the Figure 2B.

For the object detection, an experimental video was divided into frames first. The frames were then used as an input to YOLOv3 for droplet detection. YOLOv3 gives the probability of a droplet being present in an image and generates a bounding box around the image. This bounding box allows us to find the droplet location in the image and further can be used to calculate its velocity. The bounding box around the droplet is then used to crop only the droplet area as it is much easier to see crystals in a cropped image than in a complete image. The cropped area is then padded to increase the image size to 128 × 128. At this point, another deep-learning routine (ResNet50) was used to classify the image based on whether or not there was a crystal in the droplet.

Classification of the cropped images to detect the presence of the crystal is more challenging than droplet detection. One of the primary reasons for this difficulty is attributed to the different morphologies of KCl crystals, as shown in the Supplementary Information Figure S3.^49^ To solve these problems and to accurately detect the presence of the crystal, a parameter called alpha (α) defined as the ratio of the frames in which the crystal is seen within the droplet to the frames in which the droplet is seen was optimized. In addition to this, a ResNet50 algorithm was used to get high accuracies and F1-scores for different experimental videos. Furthermore, details regarding training process of the algorithm and output quality of the classifier in the form of confusion matrix numbers for all the experimental videos of *S* = 1.1 are provided in the Supplementary Information Table S3.^49^

#### 2.4.3 Statistical Analysis

The microfluidic device allows for statistically significant number of experiments (≥1000 experiments) under identical conditions to be conducted in comparison to classic NPLIN experiments (10–100 experiments), with low consumption of solute and solvents. The advantage of those large amounts of virtually identical experiments is the statistical significance of the obtained results, with major improvements regarding reliability over batch experiments.^50−54^ In the experiments performed in this study, the number of droplets containing crystals was divided by the total number of droplets to obtain the cumulative nucleation probability at fixed time lag. Nevertheless, the droplets generated in the microfluidic device are not exactly the same and a distribution is expected regarding droplet volumes, which are intrinsically related to the nucleation probability.^54−56^ Thus, it is essential to analyze mean droplet volumes (μ_v_), standard deviation (σ_v_), variance (σ_v_^2^), and coefficient of variance (ψ = σ_v_ / μ_v_) in all experiments to make sure volume variation will not significantly affect the nucleation probabilities. To account for the error in the nucleation probability, the Wilson’s score method was chosen to calculate statistical (95%) confidence intervals.^57^

### 2.5 Laser Irradiation Experiments

The developed microfluidic system is used to quantify NPLIN probability as a function of supersaturation, laser wavelength, laser intensity, solution filtration, and intentional doping. Table 1 offers an overview of the experimental conditions for all experiments. Supersaturated aqueous KCl solution used as the dispersed phase is prepared with two different supersaturations (*S* = 1.05 and 1.10). The cooling crystallization experiments with identical supersaturations were performed as controls for the laser irradiation experiments. In both irradiation and control experiments performed, the desired supersaturation was created by allowing the droplets to cool down from the temperature of the droplet generation zone, as illustrated in Figure 1. For the NPLIN experiments, droplets of solution were irradiated with the laser beam 8 cm after droplet generation in the temperature-controlled environment. Laser wavelengths (1064, 532, and 355 nm) commonly used in the NPLIN literature were utilized to investigate the effects of laser wavelength on the nucleation probability. Four different laser intensities (10, 25, 50, and 100 MW/cm^2^) were tested at each wavelength. Moreover, the role of impurities facilitating nanoparticle heating mechanism was examined by filtering KCl solutions through different pore size filters and intentionally doping Fe_3_O_4_ nanoparticles into the filtered solutions. Each experiment consisted of at least 1000 droplets.

**Table 1:** Overview of Experimental Conditions Used During Laser Irradiation Experiments Varying Supersaturation, Laser Wavelength, and Laser Intensity

experimental condition	value	unit
dispersed phase fluid	KCl	
dispersed phase flow rate	10	μL/min
continuous phase fluid	silicone oil	
continuous phase flow rate	100	μL/min
supersaturation ratios (*S*)	1.05, 1.1	
laser wavelengths	1064, 532, 355	nm
laser diameter	1.35	mm
laser intensity	10, 25, 50, 70, 100	MW/cm^2^
laser frequency	10	Hz

## 3 Results and Discussion

### 3.1 Droplet Characterization

The droplet length distribution was characterized via both the IR sensors and the deep-learning method for all the experiments performed, and the results are shown in the Supplementary Information Table S1.^49^ An example of the droplet length distributions for one of the experiment (*S* = 1.1, 1064 nm, 25 MW/cm^2^) in the form of histograms can be found in the Supplementary Information Figure S1.^49^ The length distribution based on histograms for both the methods employed displays no outliers, and the coefficient of variance was found to be 27 and 10%, respectively. The absence of outliers indicates that neither droplet coalescence nor breakage is taking place in the system. The length data obtained through the IR sensor yield a broader distribution and, consequently, a lower average length compared to the length distribution data from the deep-learning method. This is most likely due to the susceptibility of the IR sensor to external light sources. On the other hand, despite relying on an external light source to record the passing droplets in the capillary, the method of detecting droplet size by video microscopy coupled with deep-learning method was less prone to interference from the light source. Also, we compared a small sample of manually measured average droplet lengths (consisting of 100 droplets) with the average length data obtained from both the IR sensor and the deep-learning method. These results can be found in the Supplementary Information Table S2.^49^ The manually measured average droplet length closely matched the data obtained from the deep-learning method, reinforcing the conclusion that the deep-learning method provides more accurate droplet length data than the IR sensor. Consequently, further calculations were performed using the average length data obtained from the deep-learning method.

Droplet volume variation affects nucleation probability distribution since nucleation rates and detection times are intrinsically related to the volume of the crystallizer.^54^ Moreover in the NPLIN literature, the sample volume exposed to the laser has a significant effect on the nucleation rate of NPLIN according to Alexander and Camp.^16^ Thus, characterization of the droplet size is essential for robust statistics in studying NPLIN through microfluidics.

When comparing all the experiments performed under *S* = 1.05 and 1.10, we observed that the volume of droplets created varied. This variation was caused by slightly different inner capillaries used, with capillary diameters varying between 300 and 400 μm. The variation of capillary diameters across experiments was unavoidable in the experiments as the capillaries were fragile. They were replaced several times due to breakage while assembling the setup. Despite the error bars of volume distributions overlapping, the average volume changed significantly between experiments (more information is provided in the Supplementary Information Figure S2 A,C.^49^

**Figure 3:**
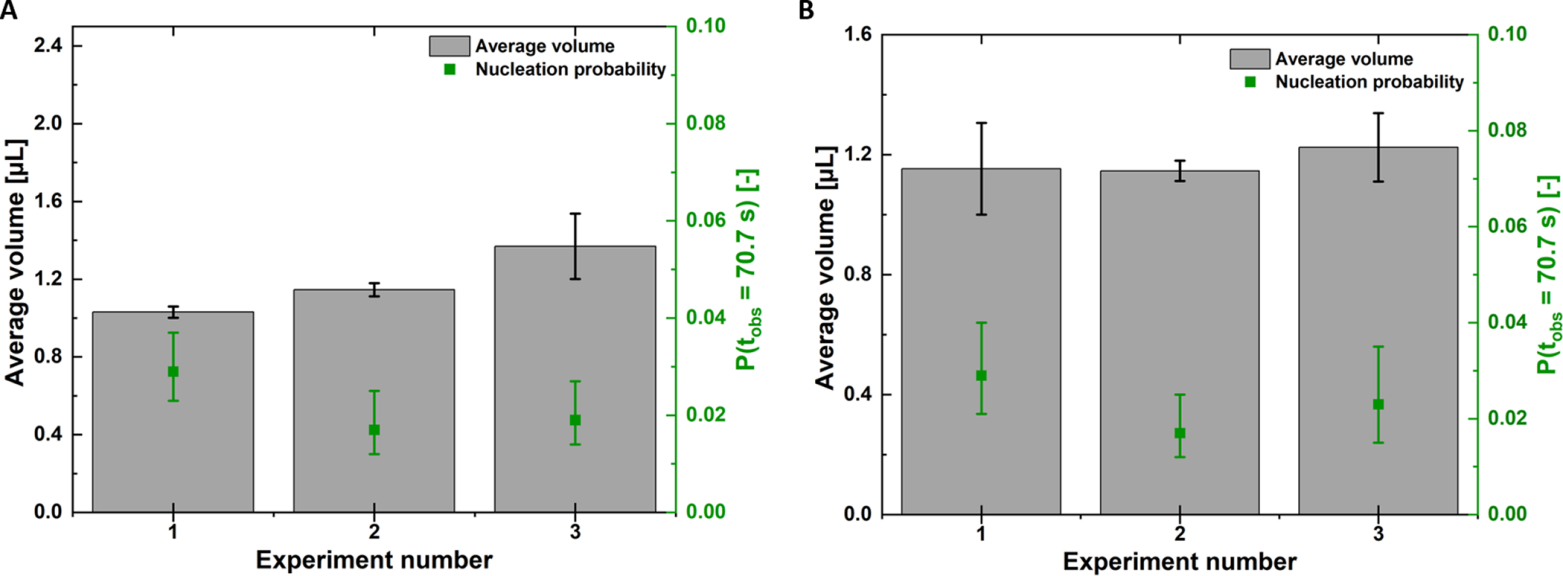
Nucleation probabilities and average droplet volumes for cooling experiments. (A) Results are shown for three distinct average droplet volumes conducted at *S* = 1.1 to evaluate the impact of average droplet volume on measured cumulative nucleation probabilities with a fixed time lag of 70.7 s. Here time lag refers to the time between laser irradiation and detection of crystals within the droplets. (B) Average droplet volumes and nucleation probabilities for three different cooling experiments conducted at *S* = 1.1 are presented to assess the experimental reliability of the developed microfluidic system.

To test whether the measured nucleation probabilities were dominated by unavoidable volume variations between experiments, we performed three independent cooling experiments where the mean droplet volume was intentionally altered. Figure 3A shows the nucleation probability of three droplet populations with varying mean volume at *S* = 1.1. We ensured that the variation in mean droplet volume in Figure 3A was similar to the experiments reported. No significant differences in the measured nucleation probability are observed in Figure 3A as the error bars overlapped for the three independent cooling experiments with three different average droplet volumes. The range of average droplet volume values changed in these three experiments was approximately the same as the variations observed in NPLIN experiments reported in this study. Hence, we conclude that the unavoidable variations in droplet volume in controlled cooling and NPLIN experiments do not significantly alter the measured nucleation probabilities.

Since the laser is irradiating the glass capillary at 10 Hz, both continuous and dispersed phase get irradiated by multiple laser pulses. As a result, the average number of pulses per droplet vary from 11 to 15 between different experiments due to variation in droplet volume and is shown in the Supplementary Information Figure S2B,D.^49^ Previous reports have demonstrated that the number of laser pulses per unit volume does not influence nucleation probabilities.^16,18,41^ Irimia et al.^18^ compared nucleation probabilities in 8 mL vials containing glycine solutions, irradiated with a single pulse and 1 min laser exposure (600 pulses) using a 1064 nm laser and found no significant difference. Nonetheless, with the presented experimental setup, it is not possible to expose droplets to a fixed number of pulses. This is a shortcoming of the developed system. A solution to this issue would be developing a microfluidic system in which droplets are temporarily stopped and then exposed to a single pulse, similar to the technique used in stop-flow lithography.^58^ However, designing and implementing such a system would require advanced microfluidic techniques and coordination between the detection system and the laser, which is beyond the scope of this current work.

### 3.2 Cooling Experiments and Repeatability

The repeatability of the microfluidic setup is checked by performing three independent cooling crystallization experiments at fixed supersaturation, *S* = 1.1, under identical conditions (laser intensity, wavelength, and cooling profile) including similar average droplet volumes. The results of these experiments are shown in the Figure 3B. No significant difference was observed in the nucleation probabilities recorded as the error bars of measured nucleation probability overlapped for the all the independent cooling experiments.

The nucleation probabilities measured for the control (cooling) experiments were lower than 3% for S = 1.10 and lower than 1.5% for *S* = 1.05. We attribute the measured non-zero nucleation probabilities to the high surface area to volume ratio of the droplets facilitating heterogeneous nucleation. Hua^41^ used comparable KCl supersaturations (from 1.06 to 1.10) and detected no nucleation for the control experiments in single-phase microfluidic NPLIN experiments. The solution flow was continuous in that study, providing a much lower surface area to volume ratio. Another potential reason is the temperature variation between experiments. Despite the fact that the lab is temperature-controlled, we cannot rule out the possibility of minute fluctuations affecting the supersaturation.

### 3.3 Laser Irradiation Experiments

**Figure 4:**
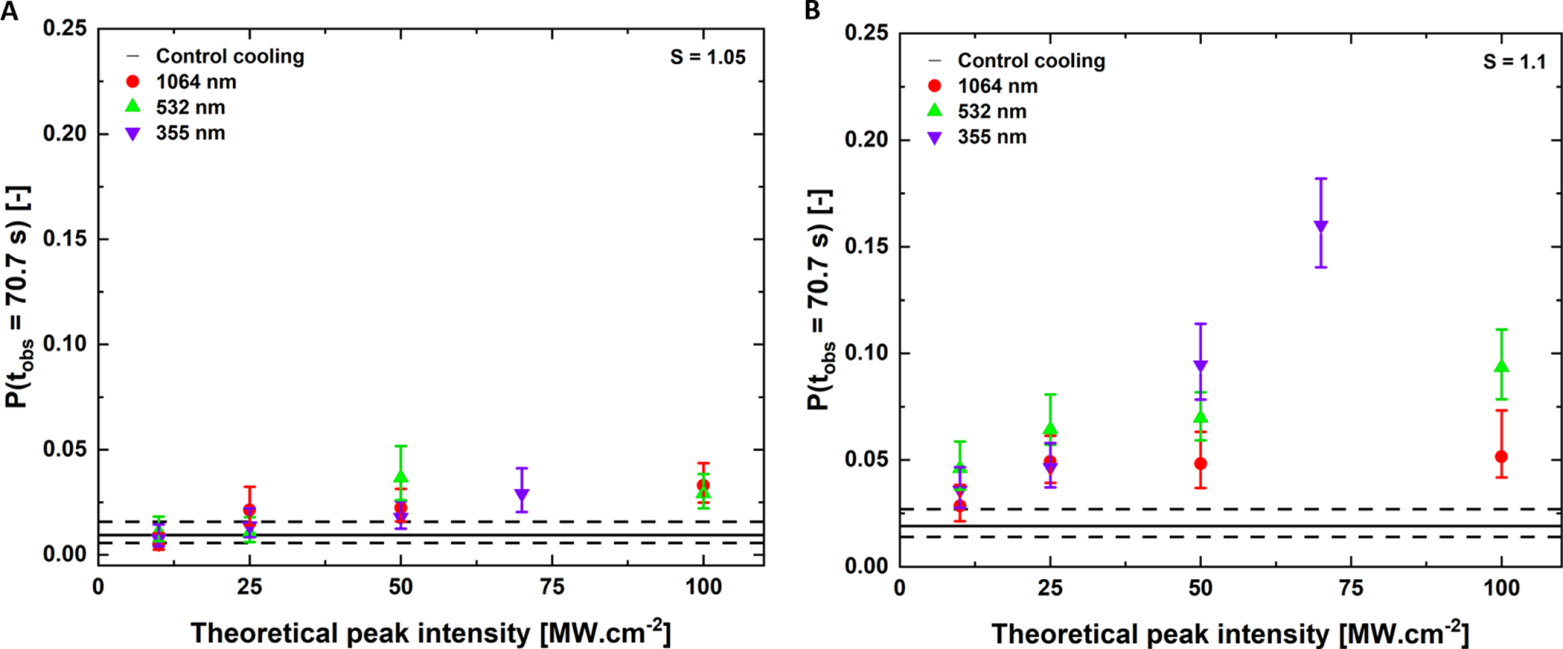
Nucleation probabilities for the experiments performed under supersaturations of (A) *S* = 1.05 and (B) *S* = 1.10, irradiated by 1064, 532, and 355 nm laser wavelengths. The dotted lines refer to the nucleation probability from control cooling experiments that serve as a reference to laser irradiation experiments. Note: an example of the nucleation probability numbers for one of the experiments (*S* = 1.1, 1064 nm, 25 MW/cm^2^), *P*(*t*_obs_ = 70.7 s) ≈ 0.049, signifies that out of 1483 droplets, there were *N* = 73 crystallization events.

Figure 4 displays the nucleation probabilities at fixed observation time for varying laser intensity (MW/cm^2^) at three different wavelengths. To facilitate quick comparison with NPLIN experiments, the results of the control (cooling) experiments included in the plot as a solid line with error bars represented as dotted line. It is worth mentioning that the average nucleation probability obtained in this study is fairly small when compared to previous reports^16,17^ for KCl. This difference is due to much lower volumes (three orders of magnitude lower) used in microfluidic scale^55^ for laser irradiation and to the substantially different detection times of crystal observation. Alexander and Camp,^16^ while conducting experiments with supersaturated KCl solution, used a fixed detection time of 20 min to check the samples for crystal formation after laser irradiation. Kacker et al.^17^ used a fixed detection time of 60 min to ensure nuclei had sufficient time to grow to a detectable size, even though after 20 min the authors observed no significant change in the nucleation probability. On the other hand, Hua et al.^41^ in their microfluidic device varied the detection times from (1–20 min) in their experiments for different combinations of supersaturation, laser intensities, and laser pulses in order to record number of crystals. In our experiments, the detection time is approximately 70.7 s and it is limited by the flow rates of dispersed and continuous phase solutions, length of the square glass capillary, and the position of the imaging system. Furthermore, the nucleation probabilities found in the cooling experiments will serve as a reference for the laser irradiation experiments. In hindsight, the low nucleation probabilities measured in our experiments can be improved by increasing the length of the capillary in order to accommodate longer detection times for crystal observation. However our attempts to work with longer capillaries were hampered by clogging issues due to poor hydrophobization.^59^ Additionally, the use of a silicone tubing in combination with 30 cm capillaries to prolong the droplets residence time caused leaks at the point where tubing was connected to the capillary tube. Therefore, capillary tubes longer than 30 cm were not used in this study.

#### 3.3.1 Effect of Laser Intensity

No laser intensity effect was observed at *S* = 1.05 (Figure 4A). For laser irradiation up to 50 MW/cm^2^ peak intensity, the measured nucleation probability was identical to the control experiments—except for 532 nm at 50 MW/cm^2^. Only for peak intensities higher than 50 MW/cm^2^ did the laser pulses increase the nucleation probability for all the wavelengths as the probabilities recorded exceeded those of the control experiments. No significant effect of wavelength on the nucleation probabilities was observed as error bars overlapped.

For *S* = 1.10 (Figure 4B), the overall trend showed that irradiation with increasingly higher laser intensities increased the nucleation probability. At 532 nm, an increase in the nucleation probability is observed for 25 and 100 MW/cm^2^, yet the probabilities for 25 and 100 MW/cm^2^ are not statistically different. The slow increase in nucleation probability for 532 and 1064 nm between 25, 50, and 100 MW/cm^2^ may indicate a saturation value above which increasing the laser intensity no longer has a direct effect on the nucleation probability. This observation is corroborated by the previous literature observations.^17^ For 355 nm, the nucleation probabilities were found to increase with increasing peak laser intensities more steeply than for other wavelengths. One possible explanation for the less steep increase observed for 532 nm and 1064 relative to 355 nm could be the local heating of the solution resulting in lower supersaturation values. Around 1064 nm, water has a slight absorption band^60,61^ which would imply some heating effect in the supersaturated solution upon laser irradiation. Previously, Irimia et al.^18^ conducted batch NPLIN experiments with supersaturated aqueous glycine solutions at a 1064 nm laser wavelength. They observed a similar local heating of the solution and identified two competing phenomena with opposite effects. The local heating of the supersaturated solution reduces the supersaturation, thereby lowering the nucleation probability. On the other hand, a temperature gradient induces mixing, which contributes to the enhancement of the apparent nucleation probability. According to the authors, the temperature effect on batch samples (8 mL) is negligible. In our study, the much lower volume of the droplets and the fact that the full droplet is irradiated eliminates the induced convective mixing effect and its influence on the nucleation probability. While this interpretation might not hold for all wavelengths, it does apply for KCl solutions between 355 and 532 nm. Within this range, the solutions show no detectable absorption bands^60,61^ despite the small ones shown by water near 355 nm.^62^ These bands are so weak that they cannot cause any significant heating effect, particularly when compared to the near-IR spectrum. Another explanation might be found in impurity heating mechanism. This mechanism revolves around the rapid heating of impurity nanoparticles, which leads to the formation of a small vapor cavity, analogous to laser-induced cavitation. In the vicinity of this cavity, the solute concentration may be enhanced, thereby promoting nucleation.^63^ Nevertheless, as these impurity particles absorb energy, a rapid temperature increase occurs in the surrounding solution, temporarily reducing local supersaturation. At lower intensity irradiation, the applied energy may not be large enough for the solution temperature to reach the vaporization temperature.^46,63,64^ A competition between heating and vapor cavity effects on supersaturation may take place at higher laser peak intensities, suggesting a threshold value for NPLIN. Previous studies in the literature have shown evidence of threshold intensities. Alexander and Camp^16^ reported a threshold for NPLIN in batch KCl solution samples, indicating its value to be practically supersaturation independent at 6.4 ± 0.5 MW/cm^2^. Kacker et al.,^17^ in batch irradiation of *S* = 1.035, 1.049, and 1.055 KCl solutions, found the threshold value to be around 0.5 MW/cm^2^ and observed 100% nucleation at laser intensity values above 5 MW/cm^2^. In this study, threshold values were found to be ≥10 MW/cm^2^ for *S* = 1.10 and ≥50 MW/cm^2^ for *S* = 1.05. The difference in threshold values is possibly due to the smaller volumes used in this study. The dynamics in a batch scale experiment differ significantly from the effects observed on the microfluidic scale.^56^ In droplet microfluidic experiments, much smaller volumes and detection times are used. Furthermore, the entire solution volume is irradiated by the laser as opposed to partial volume irradiation in batch experiments.

#### 3.3.2 Effect of Laser Wavelength

Overall, no significant wavelength effect on nucleation probability was observed. The measured nucleation probabilities followed the same trend when *S* = 1.05 (Figure 4A) solutions were irradiated with three different wavelengths. The error bars for all laser intensities overlap in Figure 4A, indicating no statistically significant wavelength effect. At *S* = 1.10 (Figure 4B), also no significant variation was observed when droplets were exposed to 1064 or 532 nm laser pulses. Even at the higher laser intensity (100 MW/cm^2^), the obtained nucleation probabilities are still considered comparable.

An exception of this general trend is nucleation probabilities measured in droplets irradiated at 355 nm for intensities ≥50 MW/cm^2^. It is noteworthy to mention that the irradiation with 355 nm proved to be experimentally challenging compared to experiments conducted with 1064 and 532 nm. Whereas with 1064 and 532 nm, it was possible to irradiate the square borosilicate capillaries with laser intensities up to 100 MW/cm^2^ for a long period of time (over 2.5 h) at 355 nm the irradiation above 70 MW/cm^2^ resulted in broken capillaries in a matter of minutes. This observed effect hindered data collection at these higher laser intensity values so the highest applied laser intensity for 355 nm was 70 MW/cm^2^. The nucleation probabilities under 355 nm were approximately two times higher than for 1064 and 532 nm above peak laser intensities (≥50 MW/cm^2^). Slightly higher nucleation probabilities upon irradiation of KCl solutions with 355 nm as compared to irradiation with 1064 and 532 nm have been reported before for all laser intensities.^17^ However, in our studies, the effect was only observed for the higher laser intensities (≥50 MW/cm^2^). The wavelength effect observed in our study could also be attributed to the photochemical effect induced by UV light irradiation, potentially heating smaller impurity particles in a manner distinct from the nanoparticle heating mechanism.^17,65^ However, a definitive verification of this hypothesis extends beyond the scope of the current work.

#### 3.3.3 Effect of Supersaturation

NPLIN probability has been reported to increase with increasing supersaturation in macroscopic NPLIN experiments.^22^ Comparing panels A and B in Figure 4 shows how supersaturation influences nucleation probability. Overall, nucleation probabilities for all theoretical peak intensities are higher for *S* = 1.10 compared to *S* = 1.05. For *S* = 1.10, irradiation with 10 MW/cm^2^ has a higher NPLIN probability than the control indicated with dotted lines. On the other hand, for *S* = 1.05, irradiation at intensities up to ≥50 MW/cm^2^, for 1064 and 355 nm, are still inefficient in triggering NPLIN where the measured nucleation probabilities are similar to control experiments.

### 3.4 Comments on NPLIN Mechanisms

#### 3.4.1 Dielectric Polarization Model

We first investigate the ability of dielectric polarization (DP) model hypothesis to explain our experimental findings of nucleation probabilities at different theoretical peak intensities at each wavelength, for both of the studied supersaturations. The DP model can be interpreted such that the number of crystals is directly proportional to the peak laser intensity (eq 1). Equation 2 can then be used to describe the nucleation probability,^16^ where *I* is the laser peak intensity and *m* is the lability factor. The lability factor in the NPLIN literature describes the ease with which a system nucleates and is thought to be specific for each solute.^22^ In this study, *t*_obs_ denoted fixed observation time taken as *t*_obs_ = 70.7 s. Analysis of the data in the DP model thus requires the determination of the lability factor.
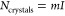
1
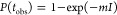
2

However, the relationships in eqs 1 and 2 fail to accurately describe a peak laser intensity threshold for NPLIN to occur, encountered in experimental data.^22^ Therefore, eqs 1 and 2 are generally adjusted to eqs 3 and 4, respectively, where *I*_o_ is the threshold theoretical peak intensity.
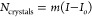
3
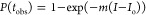
4

**Figure 5:**
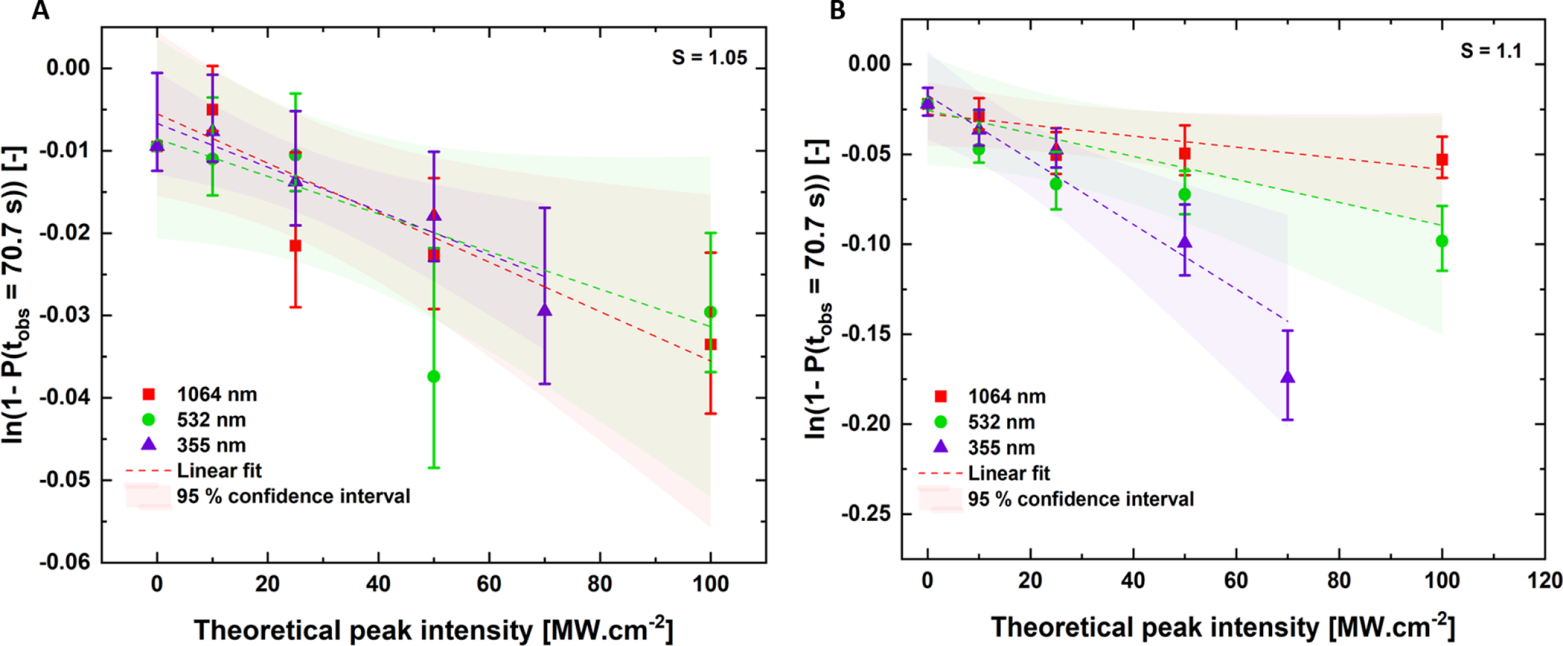
DP model semi logarithm straight line fits with 95% confidence interval prediction for all the wavelengths of experimental data under supersaturations of (A) *S* = 1.05 and (B) *S* = 1.10.

The analysis was carried out by plotting a semi-logarithm graph between 1 – *P*(*t*_obs_ = 70.7 s) and the theoretical peak intensity. The experimental data were fitted by linear regression—where *P*(*t*_obs_ = 70.7 s) is the nucleation probability at the detection time of approximately 70.7 s (Figure 5). The lability factor was then determined directly from the slope of the line, and through the intercept, threshold peak intensity *I*_o_ was calculated. The values are shown in the Table 2 with 95% confidence intervals.

**Table 2:** Overview of the Fitted Parameters, i.e., Lability (*m*) and Threshold Peak Intensity (*I*_*o*_) for Different Wavelengths for Both the Supersaturations with Uncertainities Based on 95% Confidence Intervals

supersaturation	wavelength (nm)	lability (cm^2^ MW^–1^)	threshold peak intensity (MW cm^–2^)
1.05	1064	3.00E-04 ± 2.52E-04	-18 ± 33
	532	2.28E-04 ± 2.66E-04	-37 ± 53
	355	2.66E-04 ± 1.79E-04	-25 ± 23
1.1	1064	3.09E-04 ± 7.51E-04	-89 ± 57
	532	6.40E-04 ± 9.77E-04	-40 ± 47
	355	1.80E-03 ± 1.02E-03	-9 ± 13

By analyzing the results for *S* = 1.05 in Table 2, it can be deduced that the lability factor obtained from fitting process falls within 95% confidence intervals for all laser wavelengths and hence the irradiated solutions had similar ease to nucleate. However, when the lability factor for *S* = 1.10 is examined, substantial differences can be seen between the factors determined for 1064 and 532 and 355 nm. This difference might be due to the considerably higher nucleation probabilities observed for irradiation with 355 nm at higher laser intensities as opposed to those at 1064 and 532 nm. Interestingly, the confidence interval bounds are in the same order of magnitude as the mean values for both supersaturation levels at all laser wavelengths.

From Table 2, we found that the values of lability at all the wavelengths vary by 1 or 2 orders of magnitude compared to the literature values from Ward and Alexander^22^ and Hua et al.^42^ The primary reason for this finding is likely the difference in sample volume subjected to laser irradiation. In this study, we irradiate microdroplets of μL volumes with a laser, as compared to the mL volumes reported in the literature. It is worth mentioning that in studies irradiating solutions in 10 mL vials, despite higher nucleation probabilities, the number of crystals per vial is usually 1–2. This means that, despite the larger volume of molecules and particles exposed to the laser, only one or two nuclei succeed in growing to detectable sizes. Considering the much lower volumes in our work, the likelihood of nucleation significantly diminishes. Therefore, to initiate the crystallization process in these smaller droplets, a higher intensity of laser irradiation is likely required to activate particles that are smaller than those that would typically induce crystallization in a larger volume. This observation strengthens the hypothesis of the nanoparticle heating mechanism discussed in the next section. Moreover, the role of interfaces should not be overlooked. In our microfluidic setup, the irradiated beam travels through the glass-oil, oil-solution interfaces twice, yet in macroscopic NPLIN experiments, the irradiation only interacts with the glass-solution interface twice. This difference in interface interaction should be taken into account when comparing results from the microfluidic and macroscopic setup.

Moreover, the threshold peak intensity values reported in Table 2 for both supersaturations display negative values, which are physically unrealistic. This discrepancy arises because the DP model does not account for background spontaneous crystallization, i.e., nucleation in the control experiments. Additionally, since the nucleation probabilities (*P*(*t* = 70.7 s)) yield low numbers, the slope of the fitted line is also low, resulting in low lability factors and negative threshold intensities. Improvements to the setup to increase *P*(*t*) and reduce background spontaneous crystallization could potentially yield better fitting results. In contrast, multiple laser intensity thresholds for supersaturated aqueous KCl systems under similar conditions were reported in the literature for batch sample irradiation^16,17,41^ as previously mentioned. From this analysis, we conclude that the DP model could not describe our experimental findings; hence, more focus was put on further experiments testing the (nano)impurities heating mechanism.

### 3.5 Solution Filtration

#### 3.5.1 Effect of Filter Pore Size

**Figure 6:**
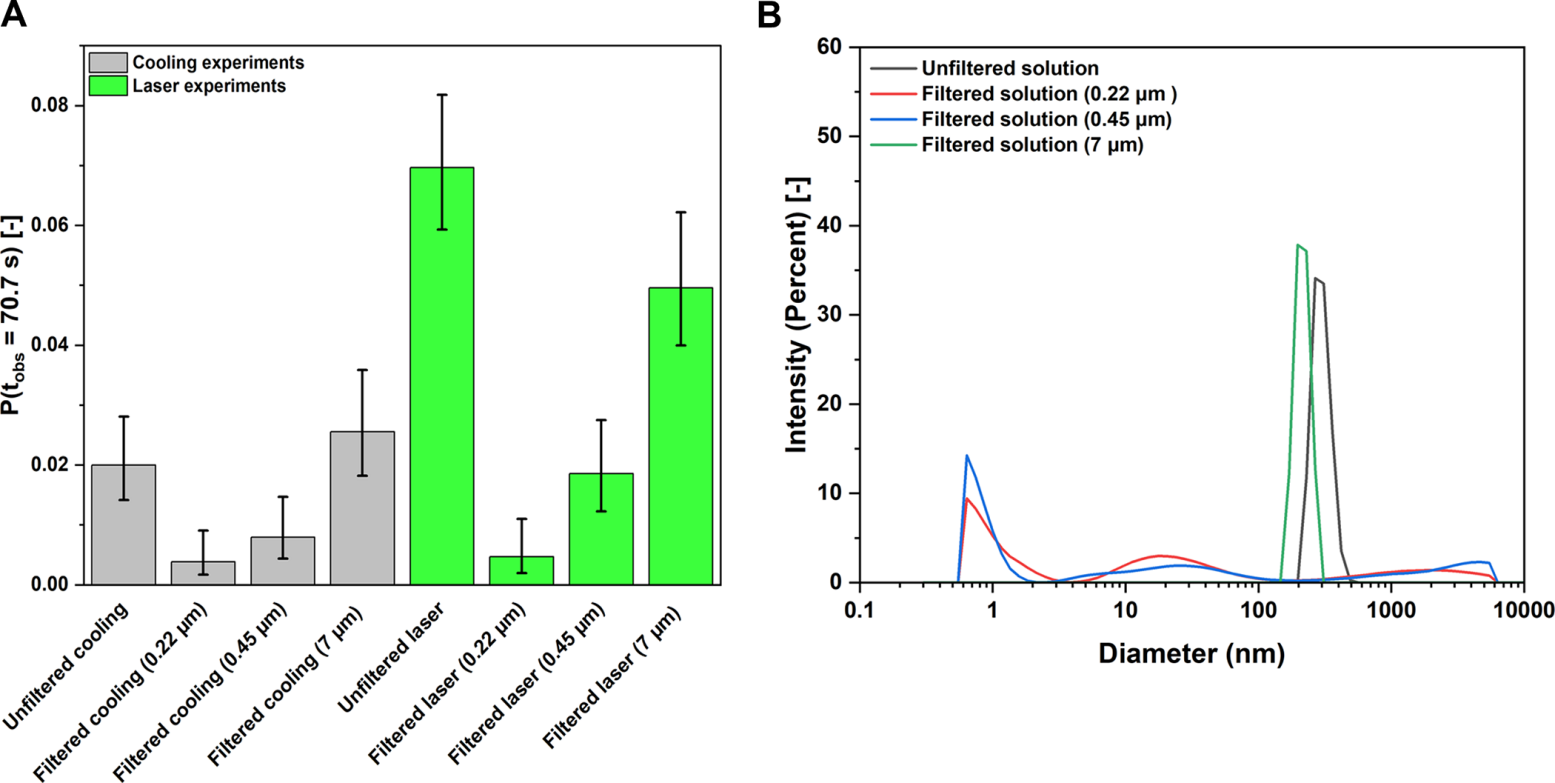
(A) Nucleation probabilities for filtered solution with different pore size diameters and unfiltered solution under *S* = 1.1 in both control cooling and laser experiments at a constant laser wavelength (532 nm) and constant theoretical peak intensity (50 MW/cm^2^) and (B) particle size distribution obtained for unfiltered KCl solution and filtered KCl solution with 0.22, 0.45, and 7 μm filters.

To investigate the influence of filtration on NPLIN probability, a series of experiments were performed with KCl solution (*S* = 1.10) with filters of different size, namely 0.22 μm (PTFE syringe filter), 0.45 μm syringe filters (PTFE syringe filter), and 7 μm paper filter (Grade-3HW, Whatman filter). The experiments were carried out using the developed microfluidic setup and included both control cooling experiments and laser irradiation experiments with incident wavelength of 532 nm and peak intensity of 50 MW/cm^2^. The nucleation probabilities obtained in these experiments are shown in the Figure 6A.

Higher nucleation probability was observed in laser experiments with an unfiltered solution as compared to a filtered solution from 0.22 to 0.45 μm pore size filters. Moreover, laser irradiation increased the nucleation probability in the unfiltered solution, while for the filtered solution, no significant difference is seen between cooling and laser experiments for 0.22 and 0.45 μm pore size filters. This observation is attributed to the presence and absence of impurities in the unfiltered and filtered solutions, respectively, which are intrinsically related to the (nano)impurity heating mechanism proposed for the NPLIN phenomena.^17,46,66^ Yet another explanation for the observed reduced nucleation probability upon filtration is the reduction of existing KCl clusters due to the high shear force produced as the fluid travels through the sub-micrometer size pores of the filter. As drag force scales with size at low Reynolds number flows,^58^ disordered clusters that are discussed in two-state nucleation theory^9^ may be broken into smaller sizes or dissolve back into the solution upon filtration. Further laser experiment results showed similar nucleation probability for both 7 μm pore size filtered and unfiltered solution, indicating the 7 μm filter was ineffective in removing nanoimpurities/nanoclusters present in the solution. This is further supported by the similar results obtained for control cooling experiments. These findings suggest that the initial presence of nanoimpurities/nanoclusters in the unfiltered solution might be larger than 0.45 μm in mean hydrodynamic diameter and could not be effectively filtered by the 7 μm pore size filter. A supportive evidence to this claim also comes from dynamic light scattering (DLS) data for these experiments as shown in the Figure 6B.

DLS was used to estimate the particle size distribution (PSD) in KCl solutions. To prevent spontaneous nucleation, the KCl solution was slightly undersaturated (*S* = 0.98). The non-negative least squares approach was used to compute the PSD from the DLS data. The measurements were performed for unfiltered KCl solution and filtered KCl solutions with different pore size filters (0.2, 0.45, 7 μm). The correlation functions and the corresponding fitted PSD for all the solutions are shown in Supplementary Information Section S6.^49^ The PSD of the unfiltered solution reveals a mean hydrodynamic diameter of 264 ± 50 nm. Upon filtration with 0.22 and 0.45 μm filters, particles at 264 ± 50 nm were eliminated, resulting in a residual population of particles ≤70 nm and ≤200 nm, respectively. Still for solutions filtered with 0.22 μm and 0.45 μm filters, peaks are seen in ≤1 nm. However, the literature reports that particle populations ≤1 nm from DLS measurements were identified as scattering from the solute and do not correspond to a true representation of the particles in solution.^46^ In contrast, filtration with a 7 μm filter produced a PSD in a similar size range to that of the unfiltered solution with a mean hydrodynamic diameter of 209 ± 14 nm. These findings suggest that the 7 μm filter was not effective in eliminating nanoimpurities or clusters and led to a nucleation probability comparable to that of the unfiltered solution. Similarly, the 0.22 and 0.45 μm filters effectively removed nanoimpurities from the unfiltered solution, thus resulting in lower nucleation probability. The obtained results in Figure 6 provide supporting evidence for the nanoparticle/impurity heating mechanism.^67^

#### 3.5.2 NPLIN Probability in Doped Solutions

**Figure 7:**
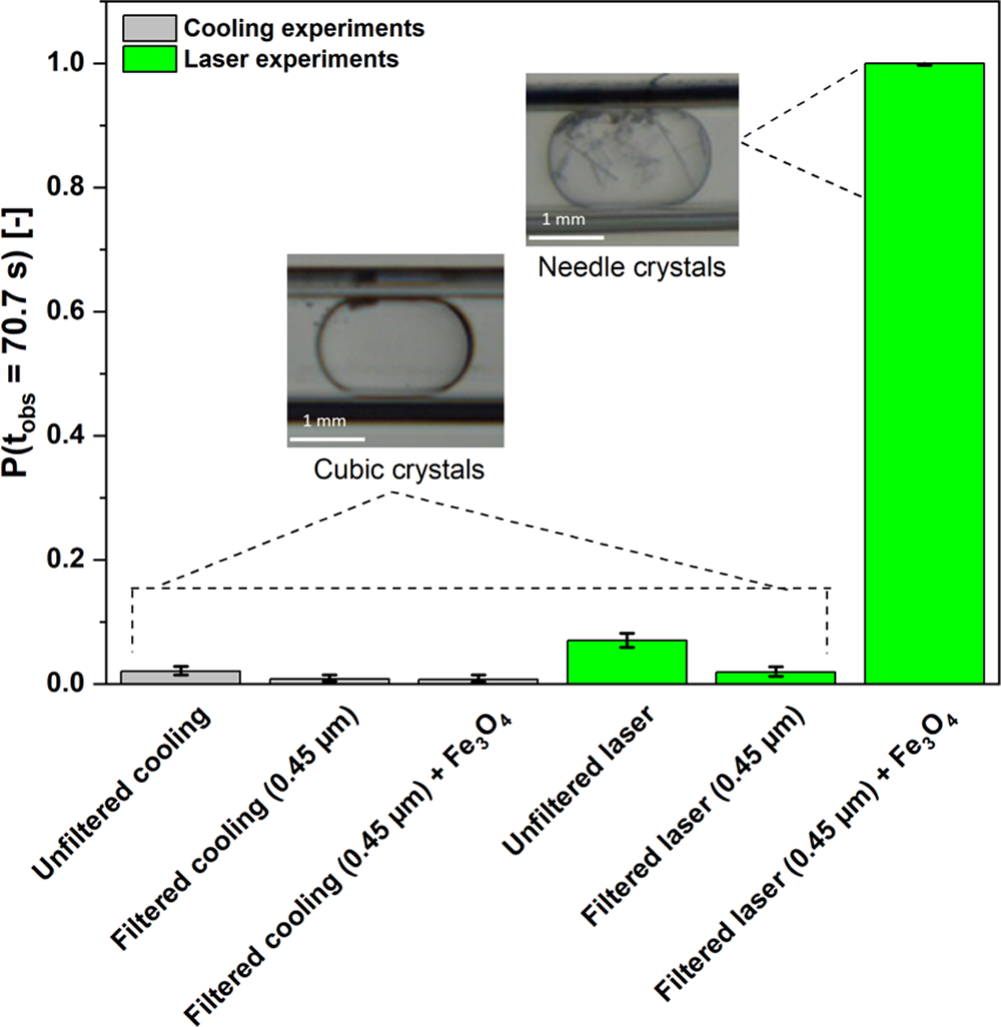
Comparison of nucleation probabilities for filtered solution along with addition of Fe_3_O_4_ nanoparticles and unfiltered solution under *S* = 1.1 in both control cooling and laser experiments at a constant laser wavelength (532 nm) and constant theoretical peak intensity (50 MW/cm^2^).

Subsequently, laser-induced nucleation experiments were performed with filtered solution (0.45 μm pore size filter) doped with Fe_3_O_4_ nanoparticles (50–100 nm nominal diameter) with a concentration of 14.6 μg/mL in solution droplets, with an incident wavelength of 532 nm and a peak intensity of 50 MW/cm^2^ to determine if addition of nanoparticles can enhance NPLIN nucleation probability by laser-impurity interaction. The results in Figure 7 show a nucleation probability of 100%, with multiple crystals formed per droplet, compared to unfiltered and filtered laser experiments where mostly a single crystal per droplet was observed. One possible explanation for the presence of multiple crystals per droplet is the high number of nucleation sites that are active within the droplet in the form of dopant nanoparticles. Another factor that could contribute to this phenomenon is the use of multiple laser shots (10–15) per droplet, which could trigger secondary nucleation events within the droplet. Similarly, control cooling experiments performed with filtered solution doped with Fe_3_O_4_ nanoparticles resulted in a nucleation probability comparable to unfiltered and filtered solution (0.45 μm pore size filter) cooling experiments results. These findings provide additional support for the observations derived from laser experiments, indicating that the dopant nanoparticles may not be intrinsically enhancing the nucleation process through heterogeneous nucleation. Instead, the laser-nanoparticle interaction within the droplet is likely the primary factor contributing to the observed nucleation behavior.

The increase in nucleation probability for doping solutions could be attributed to the fact that Fe_3_O_4_ nanoparticles exhibit a specific absorption efficiency when exposed to 532 nm laser light depending on the size of the nanoparticles. This allows us to estimate the energy absorbed by the nanoparticle from the laser. Quantitative information about specific absorption to size of the Fe_3_O_4_ nanoparticle can be found in the work from Nagalingam et al.^68^ This energy can further be used to vaporize the surrounding liquid and create a vapor bubble. To calculate the size of the vapor bubble formed from laser irradiation, we could use simple thermodynamic calculations, assuming that one laser shot on one nanoparticle produces one vapor bubble.^26^ However, the DLS result of the filtered doped solution gave a PSD with mean hydrodynamic diameter of 465 ± 33 nm, revealing that iron oxide nanoparticles are agglomerated within the supersaturated solution, a commonly encountered problem in high ionic strength solutions.^69^ Consequently, treating the agglomerated particle as a single particle may not be entirely accurate, given that the complex nature of agglomeration leads to modifications in the nanoparticles properties, including variation in its optical characteristics.^69^ These differences affect the way the agglomerated particle interacts with laser light. Therefore, accurately estimating the bubble size for the agglomerated system is beyond the scope of this paper.

At this stage, we hypothesize that upon laser irradiation of the filtered doped solution, there might be numerous vapor bubbles that would eventually merge into a larger bubble compared to the bubble size that would have been obtained in an unfiltered solution. The maximum size of the bubble, as predicted by Hidman et al.^63^ numerically and by Nagalingam et al.^68^ combining experiments and numerics, would lead to a higher local supersaturation around the vapor–liquid interface. This increased local supersaturation could accelerate the nucleation process and could explain the much higher nucleation probabilities observed in doped solutions compared to unfiltered solutions upon laser irradiation. Moreover, the morphology of crystals within the droplets obtained in laser-irradiated doped solutions, as compared to unfiltered solutions, supports this hypothesis. In nearly every droplet containing nanoparticles, we observed multiple needle-shaped crystals, suggesting creation of a high degree of local supersaturation upon laser irradiation. This is consistent with reports on the tendency for needle-shaped KCl crystals to form in higher bulk supersaturation conditions. In contrast, the presence of mostly cubic KCl crystals in almost every droplet of laser-irradiated unfiltered solutions indicates relatively lower local supersaturation levels, aligning with the typical formation of cubic crystals in lower bulk supersaturation environments.^70,71^

From the perspective of impurity heating mechanism hypothesis, the reduction of the nucleation probability in filtered solutions and enhancement of nucleation probability in dopant solutions may be interpreted as a consequence of the reduced and enhanced interaction respectively between the laser and the impurities. Several authors have provided evidence to substantiate this claim. Javid et al.^66^ performed NPLIN experiments with filtered and unfiltered aqueous glycine solutions, in which they observed a suppression of nucleation probability in filtered solution and a change in the favored polymorphic form for filtered glycine solutions compared to unfiltered solutions. Ward et al.^46^ investigated the effect of intentionally added impurities of Fe_3_O_4_ nanoparticles and polyethylene glycol surfactant on the nucleation probability and crystal count in aqueous NH_4_Cl solutions. These authors have seen that filtration significantly decreases the nucleation probability, which could again be increased to the initial (unfiltered) levels by doping the solution with the Fe_3_O_4_ nanoparticles. The same authors also found lower nucleation probabilities of CO_2_ bubbles in filtered carbonated sucrose solutions.^26^ Similarly, NPLIN experiments were carried out in filtered and unfiltered aqueous KCl solutions by Kacker et al.^17^ in which they concluded that NPLIN depends on the presence of impurities in solution. Although the observations of our experiments, along with previous claims in the literature, strengthen the evidence of a dependence of NPLIN on the presence of (nano)impurities, additional research is still required to deliver a definitive statement on the role of impurities in the induction of nucleation in NPLIN research. Further studies exploring the effects of various nanoparticles with different absorption efficiency, nanoparticle concentration and their sizes on laser intensity threshold, and nucleation probability in supersaturated solutions may provide further insights into the mechanism of NPLIN.

## 4 Conclusions

A droplet-based microfluidic system tailor designed for NPLIN studies, in combination with a fully automated droplet and crystal count monitoring system using a deep-learning method, is reported for the first time to study NPLIN. The design addresses a major criticism on the NPLIN literature, i.e., the lack of large data sets due to manually intensive nature of bulk NPLIN experiments. Variations in the form of experimental conditions such as supersaturation, laser wavelength, laser intensity, and the effect of solution filtration are studied using aqueous KCl solutions to quantify their influence on NPLIN kinetics and draw parallels with the proposed underlying NPLIN mechanisms in the literature. With respect to the laser peak intensity experiments for *S* = 1.05, laser irradiation was only proven to be effective at laser intensities ≥50 MW/cm^2^. Notably, no significant difference in nucleation probabilities as function of the laser peak intensity was found at any wavelength for this supersaturation. For *S* = 1.10, the irradiation was already seen to be effective at laser intensities ≥10 MW/cm^2^. As for the influence of laser wavelength, besides larger values obtained with irradiation of *S* = 1.10 with 355 nm at laser intensities ≥50 MW/cm^2^, no significant wavelength effect was observed. These observations are speculated to be caused by non-linear absorption of the light by the impurities within the solution. Finally, concerning the effect of supersaturation, it was evident that a higher supersaturation resulted in a higher nucleation probability. The dielectric polarization model could not describe the measured nucleation probabilities with different wavelengths as the lability parameters and threshold peak intensities calculated are inconsistent with the literature and physically unrealistic. Solution filtration with pore size less than 7 μm suppressed the NPLIN probabilities. The addition of Fe_3_O_4_ nanoparticles to the filtered solution enhanced the nucleation probabilities and altered the morphology of emerging crystals. These results highlight the role of the impurities in the solution and reinforce the nanoparticle/impurity heating mechanism hypothesis for NPLIN. It is noteworthy that the droplet microfluidic setup presented here exhibits some limitations arising from both the small volume of the droplets and the high surface area-to-volume ratio. As a result, it yields low nucleation probabilities and increases the background spontaneous nucleation (control experiments). Experimental efforts to increase the measured NPLIN probabilities by increasing supersaturation resulted in clogging of capillary downstream. Experiments at higher observation times or higher laser intensities to measure higher NPLIN probabilities were experimentally challenging due to fragile nature of the capillaries. Despite these limitations, the proposed setup may offer advantages such as statistical accuracy and ability to automation, provided that droplet microfluidics setup is re-designed to reach higher observation times or higher laser intensities.

## Supporting Information Available

Hydrophobization protocol, estimation of cooling length, IR sensors vs deep learning: a comparison, droplet characterization results, deep-learning method, and DLS results (see ref (49)) (PDF)
