# Supplementary material for: Design and Validation of a Droplet-based Microfluidic System To Study Non-Photochemical Laser-Induced Nucleation of Potassium Chloride Solutions
Source: Cryst Growth Des. 2023 Jul 19;23(8):6067–80. doi: 10.1021/acs.cgd.3c00591 (PMC10401630; doi:10.1021/acs.cgd.3c00591)
Supplement: Supplementary file 1 — cg3c00591_si_001.pdf [file cg3c00591_si_001.pdf]

Supplementary Information: Design and validation of a droplet-based microfluidic system to study Non-Photochemical laser induced nucleation of potassium chloride solutions

Vikram Korede,† Frederico Marques Penha,‡ Vincent de Munck,† Lotte Stam,† Thomas Dubbelman,† Nagaraj Nagalingam,† Maheswari Gutta,† PingPing Cui,¶ Daniel Irimia,† Antoine E.D.M. van der Heijden,† Herman J.M. Kramer,† and Hüseyin Burak Eral*,†

†*Process & Energy Department, Delft University of Technology, Leeghwaterstraat 39, 2628 CB Delft, The Netherlands*

‡*Department of Chemical Engineering, KTH Royal Institute of Technology, Teknikringen 42, 114-28 Stockholm, Sweden*

¶*School of Chemical Engineering and Technology, State Key Laboratory of Chemical Engineering, Tianjin University, Tianjin 300072, People's Republic of China.*

E-mail: h.b.eral@tudelft.nl

1 Hydrophobization protocol

The following steps were taken to hydrophobically coat the 30 cm capillaries used in the microfluidic set-up for NPLIN experiments:

- Flush the inside of the capillary with 10 mL of 0.1M Sodium Hydroxide (NaOH) solution.
- Rinse the the inside of the capillary with 20 mL of ultra pure water.
- Dry the capillary externally (if wet) using a paper towel.
- Dry the inside of the capillary with pressurized nitrogen gas.
- Add 16 μL of trichloro(1H,1H,2H,2H-perfluoroctyl)-silane to a 1.5 mL glass vial and close it.
- Insert the capillary into the 1.5 mL glass vial through a hole in its cap.
- Place the vial with the inserted capillary into a desiccator.
- Close off the desiccator in the fume hood, reduce the pressure to $<$30 mbar and let it rest overnight ($>$ 12 hours).
- Carefully return the desiccator to atmospheric pressure, open it in the fume hood and let it sit for a few minutes for any residual trichloro(1H,1H,2H,2H-perfluoroctyl)-silane to evaporate.
- Wipe the capillary externally with isopropanol, repeat with acetone and flush with pressurized nitrogen gas to ensure any residue of trichloro(1H,1H,2H,2H-perfluoroctyl)-silane is removed from the capillary.

2 Estimation of cooling length

The IR temperature sensor was implemented in the system to study droplet temperature before the laser exposure location to make sure the droplets have reached ambient temperature. This is important, because only then the supersaturation of the KCl solutions in the droplets will have the desired value. To this end, a very simplified estimation of the cooling length was determined from the formula for thermal diffusion distance and thermal diffusion time.

$$x = \sqrt{2\alpha t} \tag{1}$$

$$t = \frac{L^2}{2\alpha} \tag{2}$$

where x is the distance in mm at which droplets attain the ambient temperature (25°C) within the capillary, α is the thermal diffusivity of water at ambient temperature in m^2/s, t is the time in seconds it takes for the droplets to reach ambient temperature, L is the length of the capillary in mm. However, the analysis considered here is an approximation method to calculate the temperature profile based on the following assumptions.

- Assuming that capillary tube is filled with only one fluid which has the physical properties of water at 25°C.
- Temperature in the capillary tube is assumed to be the same along the entire length of the tube and the heat only flows in one direction (axially) and is not affected by the radial direction or outer part of the capillary. This means that the temperature profile is assumed to be linear along the capillary. It is an approximation method to calculate the temperature profile and time taken to reach final temperature.
- Calculations of the temperature profile and time taken to reach the final temperature are performed under steady state conditions, assuming that the ambient temperature is constant.

Using the equations and assumptions above, the cooling length was estimated to be around 15.6 mm. The actual cooling length will be slightly different, because both conduction through the wall hindering heat transfer and convection in the air around the capillary promoting heat transfer have not been taken into account. Although the actual cooling length might deviate from the calculated value because of the oversimplification, it is safe to assume that the droplets have cooled down to room temperature before reaching the laser exposure location 8 cm into the capillary.

3 IR Sensors vs Deep Learning: A Comparison

The droplet length distribution for all experiments performed, characterized using both IR sensors and the deep learning method, is provided in Table SI. To illustrate the results, Figure SI provides a representative histogram of the droplet length distribution from a single experiment (S = 1.1, 1064 nm, 25 MW/cm^2). Additionally, Table SII presents a comparison of the average droplet lengths obtained from the IR sensor, deep learning method, and manual measurement. The approximate volume of the droplets per experimental video was obtained by estimating the individual length of each droplet from its bounding box and multiplying the length obtained with the dimensions of the square capillary (0.9*0.9 mm^2). The average volume per experimental video was then found by finding the mean of the individual droplet volumes.

4 Droplet characterization results

As discussed in the main article, the average length results obtained from deep learning method were found to be reliable in comparision to IR sensors results. To further show the comparision between individual experiments, Droplet volume distributions obtained for all the experiments under S = 1.05 & 1.10 from

Table SI: Comparision of droplet length distribution characterized via both the IR sensors and the deep learning method for all the experiments performed

Method	Supersaturation (S)	Wavelength (nm)	Laser Intensity (MW/cm^2)	Average length (mm)	Average volume (μ_v) (μL)	Average volume variation (σ_v)	Coefficient of variation ($\psi = \sigma_v/\mu_v$)	Average pulses per droplet (#)	Average pulses per droplet ($\sigma_{\#}$))
Deep learning method	1.05	Blank	0	1.453	1.177	0.084	0.071	12.969	1.011
		1064	10	1.619	1.311	0.061	0.046	13.478	0.848
		1064	25	1.535	1.243	0.093	0.075	14.365	1.202
		1064	50	1.519	1.230	0.175	0.142	13.091	1.414
		1064	100	1.554	1.258	0.145	0.115	13.347	1.301
		532	10	1.500	1.215	0.101	0.083	12.892	1.002
		532	25	1.409	1.141	0.060	0.052	13.595	1.123
		532	50	1.702	1.378	0.163	0.118	14.805	1.657
		532	100	1.465	1.186	0.063	0.053	13.016	0.886
		355	10	1.865	1.511	0.092	0.061	14.315	1.06
		355	25	1.833	1.485	0.120	0.081	14.391	1.121
		355	50	1.509	1.222	0.105	0.086	12.423	1.010
		355	70	1.438	1.165	0.0571	0.049	12.510	0.906
	1.1	Blank	0	1.415	1.146	0.035	0.030	13.394	0.776
		1064	10	1.531	1.240	0.145	0.117	12.480	1.280
		1064	25	1.553	1.258	0.126	0.100	13.333	1.069
		1064	50	1.464	1.186	0.115	0.097	13.045	0.951
		1064	100	1.561	1.264	0.109	0.086	12.626	0.957
		532	10	1.535	1.243	0.130	0.104	13.680	1.792
		532	25	1.696	1.373	0.083	0.060	14.907	0.976
		532	50	1.299	1.052	0.029	0.027	9.863	0.783
		532	100	1.479	1.198	0.127	0.106	10.927	1.335
		355	10	1.528	1.238	0.100	0.081	12.731	1.594
		355	25	1.469	1.190	0.091	0.076	12.649	0.960
		355	50	1.498	1.213	0.063	0.052	13.505	0.985
		355	70	1.540	1.248	0.033	0.027	13.201	0.680
	Supersaturation (S)	Wavelength (nm)	Laser Intensity (MW/cm^2)	Average length (mm)	Average volume (μ_v) (μL)	Average volume variation (σ_v)	Coefficient of variation ($\psi = \sigma_v/\mu_v$)	Average pulses per droplet (#)	Average pulses per droplet ($\sigma_{\#}$))
Infrared sensors (IR)	1.05	Blank	0	1.197	0.897	0.1695	0.188	10.114	1.057
		1064	10	1.183	0.959	0.091	0.095	10.456	0.751
		1064	25	1.549	1.254	0.157	0.125	12.947	1.168
		1064	50	1.301	1.023	0.273	0.267	11.087	1.610
		1064	100	1.275	1.032	0.285	0.276	11.053	1.656
		532	10	1.176	0.952	0.195	0.205	10.371	1.225
		532	25	1.270	1.029	0.135	0.131	9.560	0.900
		532	50	1.968	1.594	0.37	0.232	13.914	2.136
		532	100	1.437	1.164	0.143	0.122	9.887	1.450
		355	10	1.581	1.280	0.170	0.133	11.792	1.127
		355	25	1.608	1.303	0.217	0.166	11.806	1.272
		355	50	1.191	0.965	0.083	0.086	10.105	0.996
		355	70	1.225	0.992	0.368	0.371	10.587	1.995
	1.1	Blank	0	1.280	1.037	0.095	0.091	10.971	1.121
		1064	10	1.428	1.157	0.609	0.526	11.050	3.151
		1064	25	1.170	0.948	0.256	0.270	10.467	1.451
		1064	50	1.024	0.829	0.175	0.211	9.934	1.049
		1064	100	1.179	0.955	0.173	0.182	10.368	1.132
		532	10	1.428	1.280	1.037	0.196	0.189	10.939
		532	25	1.170	1.730	1.401	0.141	0.100	12.664
		532	50*	-	-	-	-	-	-
		532	100	1.179	1.254	1.016	0.303	0.298	10.771
		355	10	1.306	1.058	0.516	0.487	11.025	2.760
		355	25	1.345	1.089	0.581	0.533	10.934	2.990
		355	50	1.456	1.179	0.105	0.089	11.369	0.830
		355	70	1.223	0.991	0.074	0.075	10.751	0.679

IR sensors data was not available for the experiment with * sign.

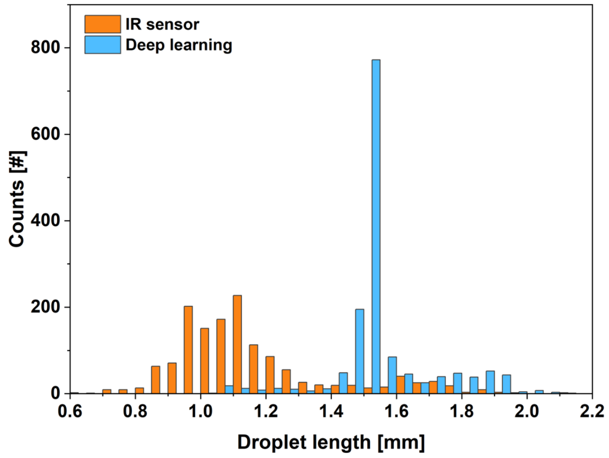

Figure SI: Comparison of the droplet length distribution using IR and deep learning methods.

Table SII: Table comparing average droplet length measurements taken via three different methods.

**Measurement method (100 readings)**	**Average droplet length (mm)**
IR sensor	1.526
Deep learning	1.812
Manual	1.833

deep learning method are shown in Figure SII A & C respectively. At each wavelength, the measurements are portrayed in order of increasing laser intensity. Similarly pulses per droplet distribution are shown in SII B & D respectively.

5 Deep learning method

As described in the deep learning section of the article, the classification of cropped images to detect the presence of a crystal is more challenging than droplet detection. One primary reason for this difficulty is the different morphologies of KCl crystals, as shown in Figure SIII. Some images of droplets, such as SIIIc, SIIId, and SIIIe, display transparent crystals that are potentially difficult to detect due to poor contrast. In addition, a few needle-shaped crystals SIIIb were also observed. Moreover, there might be images of droplets with impurities present on the outside of the capillary, which might also be classified as a crystal. Therefore, it is crucial to train the algorithm sufficiently well to robustly detect different morphologies of KCl crystals automatically.

5.1 Training information

The training of the images was not performed on experimental videos with 1.05 supersaturation because the crystals were too small for the algorithm to detect. Consequently, a manual count of droplets containing crystals was provided for 1.05 supersaturation experimental videos to estimate nucleation probabilities and for further data analysis. On the other hand, the training of the images was performed through supervised learning, i.e., hand-labeling 777 images from different videos with 1.1 supersaturation. The images were carefully chosen to ensure they were challenging to categorize and could be easily misclassified. Selecting these types of images is crucial for training the model, as the more difficult and complex the samples, the more robust the training process. The training data was labeled with 438 images of droplets containing crystals and 339 images of empty droplets. The binary classification task was performed using the ResNet50 architecture with pre-trained weights and five-fold cross-validation. Apart from the training data, 46 images were set aside as test data to validate the model's performance. The test data contained 16 images of droplets without a crystal, all of which were predicted correctly. However, 4 of the 26 images of droplets with crystals were incorrectly predicted, resulting in an accuracy of 91.17% on the test data.

5.2 Results

Once the model was trained using supervised learning, different combinations of NPLIN experiments recorded as videos were run through the algorithm one at a time to count the droplets with crystals automatically. At this point, the output quality of the classifier was measured using the F1-score metric based on a confusion matrix. In machine learning, the F1-score is defined as the harmonic mean of precision and recall. The confusion matrix numbers for all the experimental combinations, along

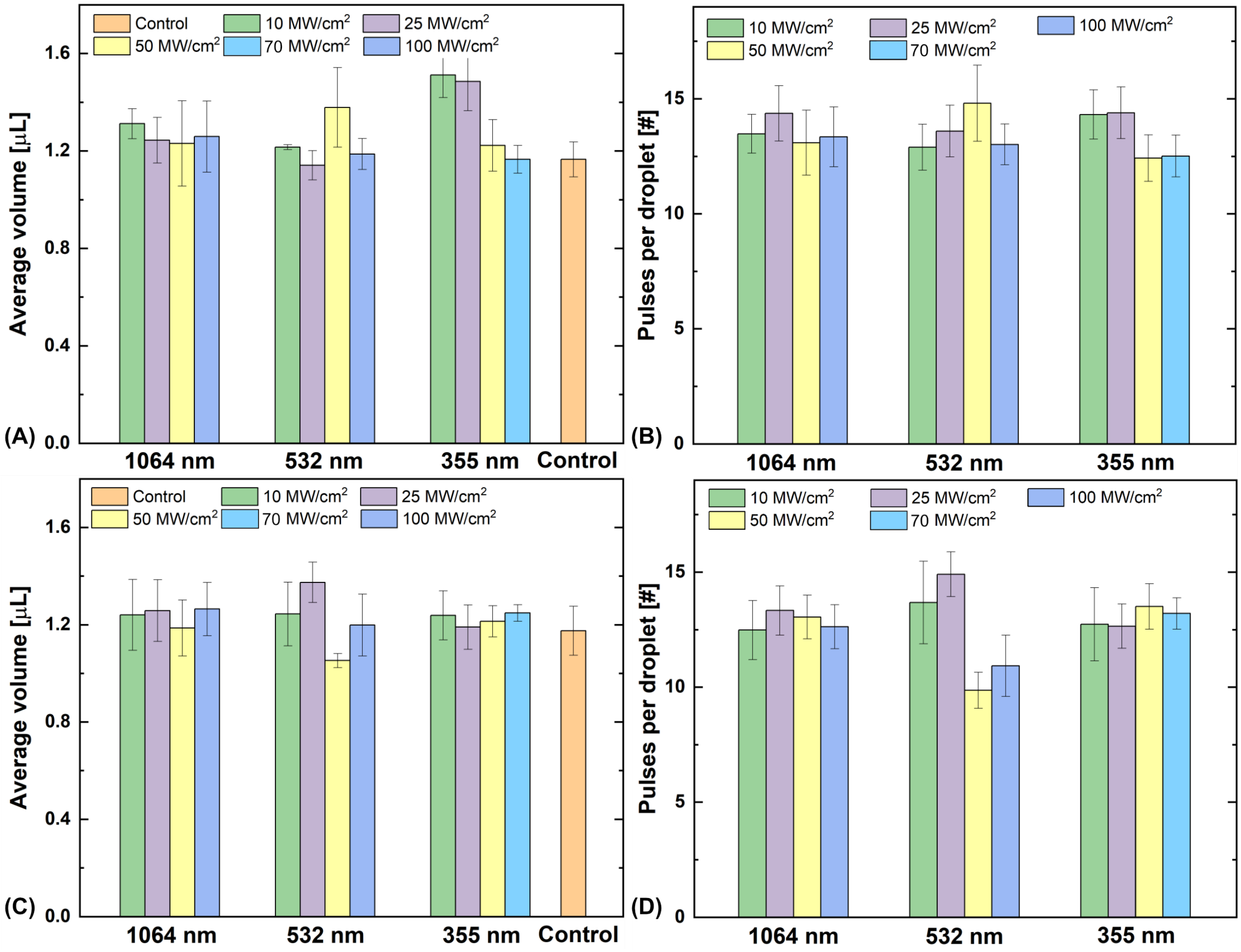

Figure SII: Droplet characterization for experiments under S = 1.05 (A & B) and S = 1.10 (C & D) showing the average droplet volume and average number of pulses per droplet for all the experiments.

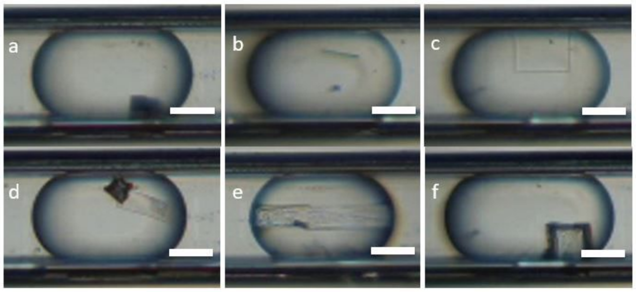

Figure SIII: Images of the different droplets having the same KCl crystal but with different morphologies. Scale bar is 1 mm for all the images.

with their F1-scores, are shown in Table SIII below. Although the accuracy of our model is very high (94.7 - 100%) between different experimental videos, there is a comparatively broad variation in the F1-scores obtained (82.7 - 100%). The main reason for this variation is that our data is skewed, i.e., there are many empty droplets compared to droplets with crystals in every experimental video. Furthermore, the wide range of F1-scores might be explained by changes in light exposure and contaminants on the capillary throughout different experiments, which result in more false negatives during crystal detection and, consequently, a lower F1-score.

6 DLS results

In the main article, Dynamic Light Scattering (DLS) results were presented for unfiltered KCl solution and filtered KCl solutions using various pore size filters (0.2 μm, 0.45 μm, 7 μm). The DLS measurements were performed at 25°C using a Malvern Panalytical Zetasizer Pro apparatus, with a 633 nm laser light source and a scattering angle of 13°. In this section, we provide a repetition of these results along with additional data for the doped solution containing Fe_3O_4 nanoparticles. Notably, the DLS count rate for filtered samples with 0.22-micron and 0.45-micron filters was quite low ($\leq$100 kcps), potentially indicating data inconsistencies. In contrast, the count rate was reliably higher ($\geq$100 kcps) for the 7-micron filtered samples (118.3 kcps), as well as for unfiltered (127.5 kcps) and doped samples (159.2 kcps), suggesting more accurate measurements.

Table SIII: The table contains the results obtained from deep learning algorithm for all the experimental combinations under 1.1 supersaturation. Output quality of the classifier was shown by confusion matrix numbers along with their F1-scores.

Super-saturation	Wavelength (nm)	Laser intensity (MW/cm2)	Number of Droplets	Droplets with crystal : Manual counting	Droplets with crystal : Automatic counting	TP	TN	FP	FN	Alpha	F1-score
1.1	blank	0	1631	32	32	32	1599	0	0	0.14	100
1.1	1064	10	1505	60	43	43	1444	1	17	0.15	82.69
1.1	1064	25	1483	79	73	73	1396	8	6	0.4	91.25
1.1	1064	50	1034	55	50	50	977	2	5	0.35	93.45
1.1	1064	100	1610	93	83	83	1517	0	10	0.3	94.31
1.1	532	10	1001*	51	-	-	-	-	-	-	-
1.1	532	25	1057	76	68	68	976	3	8	0.16	92.51
1.1	532	50	1979	138	138	138	1841	0	0	0.15	100
1.1	532	100	1229*	115	-	-	-	-	-	-	-
1.1	355	10	1478	53	53	53	1424	1	0	0.4	99.06
1.1	355	25	1571*	73	-	-	-	-	-	-	-
1.1	355	50	1046	109	99	99	928	10	10	0.07	90.82
1.1	355	70	1187	219	190	190	934	34	29	0.1	85.78

Laser specification: TP = True Positives, TN = True Negatives, FP = False Positives, FN = False Negatives, Alpha : (frames in which crystal is seen/ frames in which droplet is seen), F1-score = (2 * Precision * recall)/(Precision + recall). The quality of three experimental videos with * sign was not adequate to accurately extract data for the automatic crystal count numbers in the confusion matrix table.

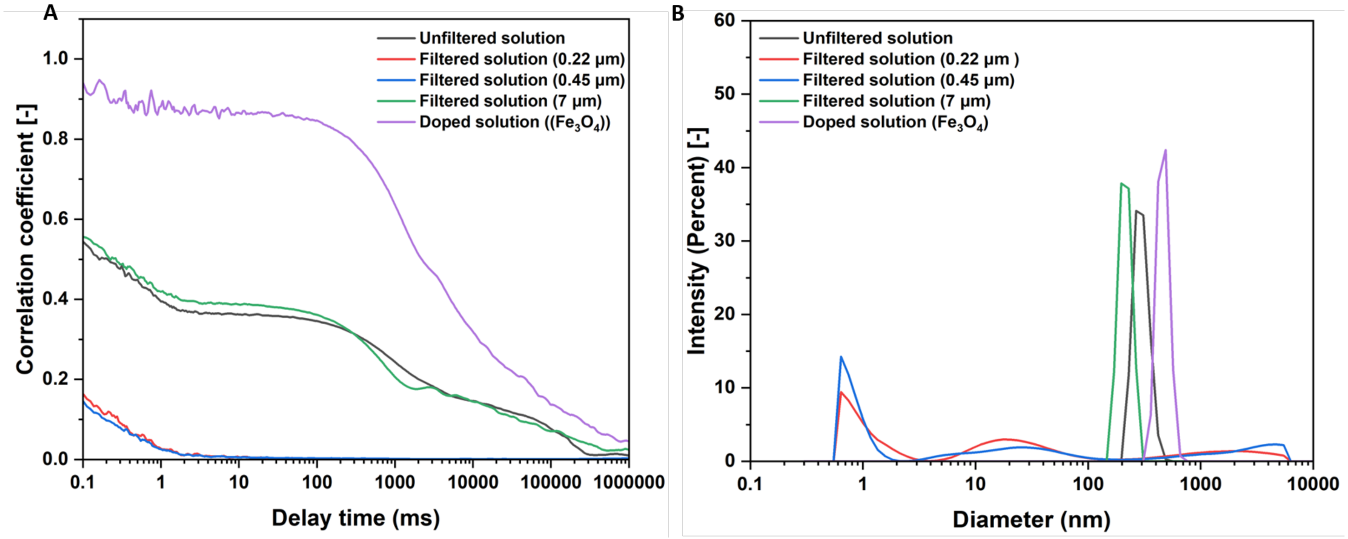

Figure SIV: (A) Cross correlation functions obtained via DLS and (B) Particle size distribution determined by fitting DLS data for unfiltered KCl solution, KCl solution filtered with 0.22 μm, 0.45 μm, and 7 μm filters, as well as the solution doped with Fe_3O_4 nanoparticles.
